# Supplementary material for: Revealing the Hidden Impacts: Insights into Biological Aging and Long-Term Effects in Pauci- and Asymptomatic COVID-19 Healthcare Workers
Source: Int J Mol Sci. 2024 Jul 24;25(15):8056. doi: 10.3390/ijms25158056 (PMC11311509; doi:10.3390/ijms25158056)
Supplement: Supplementary file 1 [file ijms-25-08056-s001.zip › ijms-3112084-supplementary.pdf]

Supplementary Material

Supplementary Tables

Supplementary Table S1. Absolute and percentage distribution of COVID-19 symptoms reported by the HCWs up to 4 weeks, from 4 to 12 weeks, over 12 weeks after diagnosis, and at the time of the visit (1-year follow-up) (n=76 HCWs).

Symptoms	Up to 4 weeks after diagnosis n, (%)	From 4 to 12 weeks after diagnosis n , (%)	Over 12 weeks after diagnosis n, (%)	P Chi2	1-year follow-up	P Chi2
**Respiratory symptoms**						
Dyspnoea	11 (14.47)	7 (9.21)	7 (9.21)	**0.4873**	5 (6.58)	**0.4217**
**Cardiovascular symptoms**						
Palpitations	11 (14.47)	7 (9.21)	6 (7.89)	**0.3761**	3 (3.95)	**0.1495**
**Neurological symptoms**						
Brain fog	10 (13.16)	7 (9.21)	3 (3.95)	**0.1316**	2 (2.63)	0.0453
Sleep disorders	20 (26.32)	11 (14.47)	10 (13.16)	**0.0668**	8 (10.53)	0.0412
Peripheral neuropathies	4 (5.26)	4 (5.26)	3 (3.95)	**0.9089**	3 (3.95)	**0.9601**
Loss of concentration	14 (18.42)	13 (17.11)	11 (14.47)	**0.8017**	9 (11.84)	**0.6858**
Memory problems	11 (14.47)	14 (18.42)	13 (17.11)	**0.8017**	11 (14.47)	**0.8833**
**Psychological or psychiatric symptoms**						
Anxiety	11 (14.47)	9 (11.84)	7 (9.21)	**0.6040**	5 (6.58)	**0.4245**
Depression	7 (9.21)	5 (6.58)	4 (5.26)	**0.6247**	0 (0.00)	Na
**Otorhinolaryngological symptoms**						
Otalgia	1 (1.32)	0 (0.00)	0 (0.00)	**0.3663**	0 (0.00)	Na
**Dermatological signs**	3 (3.95)	2 (2.63)	3 (3.95)	**0.8785**	2 (2.63)	**0.9374**
**Ocular symptoms**	11 (14.47)	5 (6.58)	4 (5.26)	**0.0947**	2 (2.63)	0.0318
**Other**	10 (13.16)	5 (6.58)	4 (5.26)	**0.1687**	3 (3.95)	**0.1280**

Abbreviations: NA= not available

Supplementary Table S2. Mean values and standard deviations of blood leukocytes DNAmAge, AgeAcc, and TL of all n=76 HCWs.

	**Age**	***Blood* leukocytes DNAmAge (years)**	***Blood leukocytes AgeAcc (years)***	***Blood leukocytes TL (T/S)***
**Mean± SD**	46.00±12.88	-2.59±3.47	1.12±4.37	1.20±0.06

Abbreviations: DNAmAge= DNA methylation Age; AgeAcc= Age acceleration; TL= telomere length.

Supplementary Table S3. Multiple linear regression analysis of the influence of leukocytes (10^9/L) and different blood cell counts, including neutrophils (10^9/L), lymphocytes (10^9/L), and monocytes (10^9/L) on blood leukocytes DNAmAge / TL.

		b	r	t value	P
**Leukocytes DNAmAge (years)**	**Leukocytes (10^9/L)**	b1 = 1.255602	r = 0.096151	t = 0.813956	0.4184
	**Neutrophils (10^9/L)**	b2 = -1.878808	r = -0.125576	t = -1.066567	0.2898
	**Lymphocytes (10^9/L)**	b3 = -4.596302	r = -0.204707	t = -1.76221	0.0823
	**Monocytes (10^9/L)**	b4 = -3.185565	r = -0.034158	t = -0.287991	0.7742
**Leukocytes TL (T/S)**	**Leukocytes (10^9/L)**	b1 = -0.082265	r = -0.248031	t = -2.15736	**0.0344**
	**Neutrophils (10^9/L)**	b2 = 0.156416	r = 0.392153	t = 3.59206	**0.0006**
	**Lymphocytes (10^9/L)**	b3 = 0.188515	r = 0.327818	t = 2.923814	**0.0046**
	**Monocytes (10^9/L)**	b4 = -0.110657	r = -0.047973	t = -0.404695	0.6869

Supplementary Table S4. Number of different tissues (blood, nasal cells, induced sputum cells) samples collected from our HCWs population, biomarkers of biological aging analyzed (DNAmAge, AgeAcc and TL), and measurements determined (mean(±SD)).

Biomarkers of biological aging	***Number of samples***	***Mean (±SD)***
DNAmAge		
Blood	76	41.59±10.50
Nasal cells	70	35.29±12.76
Induced sputum cells	19	47.00±11.70
AgeAcc		
Blood	76	-2.88±3.69
Nasal cells	70	-9.10±7.86
Induced sputum cells	19	1.74±4.71
TL		
Blood	76	1.20±0.28
Nasal cells	70	-
Induced sputum cells	18	0.89±0.42

Supplementary Table S5. Demographic and clinical characteristics of study population (n=76 HCWs).

Variable	Mean±SD	N subjects	%
Age [years]	44.64±11.75		
Gender [n (%)]			
M		22	28.95
F		54	71.05
Marital status [n (%)]			
Not married		31	40.79
Married		40	52.63
Divorced		5	6.58
Widower		0	0.00
Years of education [years]	16.67±5.57		
BMI [Kg/m2]	24.48±4.01		
Systolic blood pressure [mmHg]	123.55±13.74		
Diastolic blood pressure [mmHg]	77.43±10.55		
**EMPLOYMENT ANAMNESIS**			
Professional position: [n (%)]			
Healthcare assistant		19	25.00
Nurse		30	39.47
Doctor		19	25.00
Resident		0	0.00
Other		8	10.53
Total years of work [years]	19.74±12.65		
Years of work in the current job [years]	9.78±9.23		
Performance of night shifts [n (%)]		43	56.58
Frequency of night shifts/month			
0		33	43.42
From 1 to 4		13	17.11
>5		30	39.47
Work ability - WAI (n=68) [n (%)]			
Poor (7-27)		5	7.35
Moderate (28-36)		15	22.06
Good (37-43)		5	33.82
Excellent (44-49)		25	36.76
**PHYSIOLOGICAL ANAMNESIS AND LIFESTYLE**			

Chronic diseases [n (%)]			
0		28	36.84
1		16	21.05
≥2		32	42.11
Tobacco habit [n (%)]			
Smoker		10	13.16
Ex-smoker		13	17.11
Non-smoker		53	69.74
Pack/years [(cigarettes/20) per years of smoking]	1.63±4.26		
Alcohol consumption [n (%)]		56	73.68
Alcohol consumption [u.a./die]	106.89±155.76		
Binge drinking habit [n (%)]		1	1.32
Meals with grilled meat or pizza cooked in a wood-fired oven/year [n of meals/year]	80.63±59.72		
Frequency of fruit meals/day [n (%)]			
<2		39	51.32
>2		37	48.68
Frequency of vegetable meals/day [n (%)]			
<2		32	42.11
>2		44	57.89
IPAQ score (n=69) [n (%)]			
<700		11	15.94
≥700; ≤ 2519		20	28.99
>2520		38	55.07
Indoor pollution*			
0		52	68.42
1		19	25.00
2		5	6.58
3		0	0.00
Living area [n (%)]			
Urban/ peripheral area		50	65.79
Rural area		26	34.21
Traffic in the living area [n (%)]			
Continuous intense for a good part of the day		23	30.26

Intermittent intense		30	39.47
Scarce or absent		23	30.26
**BASIC BIOCHEMISTRY PARAMETERS**			
Leukocytes (103/ml)	6.19±1.59		
Blood red cells (103/ml)	4.70±0.43		
Platelet count (103/ml)	267.08±57.13		
Neutrophils (103/ml)	3.41±1.18		
Lymphocytes (103/ml)	2.17±0.58		
Monocytes (103/ml)	0.51±0.13		
Eosinophils (103/ml)	0.27±0.97		
Basophils (103/ml)	0.03±0.03		
Hemoglobin (g/dl)	138.18±13.79		
Glycemia (mg/dl)	92.12±14.31		
Cholesterol (mg/dl)	194.00±14.27		
Triglycerides (mg/dl)	93.11±42.18		
HDL (mg/dl)	59.85±15.58		
LDL (mg/dl)	123.04±30.74		
Creatinine (mg/dl)	1.49±6.26		
Bilirubin (umol/L)	9.60±10.58		
**LIVER FUNCTION**			
AST/GOT (U/L)	23.23±6.85		
ALT/GPT (U/L)	21.8±12.73		
GGT (U/L)	9.60±10.58		
**INFLAMMATION**			
PCR (mg/L)	5.19±3.23		
**LUNG FUNCTION**			
FEV1 (L)	3.30±0.88		
FEV1 (%)	101.76±13.54		
FVC (L)	4.10±0.92		
FVC (%)	95.56±12.68		
FEV1/VC (%)	0.82±0.06		
TLC (L)	5.55±1.26		
TLC (%)	96.47±15.70		
RV (L)	1.59±0.40		
RV (%)	107.36±23.43		
**HEART RATE**			

Mean HR	68.05±9.71
**HEART RATE VARIABILITY**	
nLF: 0.04 - 0.15 Hz	52.38±18.20
nHF: 0.15 - 0.40 Hz	47.31±18.41
LF/HF ratio	1.54±1.30
SDNN	35.16±25.45
RMSSD	36.89±34.19

*Sum of three variables: presence of a pellet or wood stove in the home used more than five times a year, extra-occupational exposure to Polycyclic Aromatic Hydrocarbons (PAH) and exposure to passive tobacco smoke.

Abbreviations: ALT= Alanine Aminotransferase; AST= Aspartate Aminotransferase; BMI= Body mass index ; FEV1= Forced Expiratory Volume in the 1st second; FVC= Forced vital capacity; GGT= Gamma glutamyl transferase; HCWs= Health Care Workers; HDL= High-density lipoproteins; HR= Heart rate; IPAQ= International Physical Activity Questionnaires; LDL= Low-density lipoproteins; nHF= normalized high frequency; nLF= normalized low frequency; PCR= C-reactive protein; RMSSD= Root mean square of successive RR interval differences; RV= Residual volume; SDNN= Standard deviation of normal-to-normal R-R intervals; TLC= Total lung capacity; WAI= Work ability index.

Supplementary Table S6. Data relating to the course of SARS-CoV-2 infection (n=76 HCWs).

Variable	Mean±SD	N subjects	%
Duration of SARS-CoV-2 infection [days]	17.81±6.03		
Diagnosis of SARS-CoV-2 pneumonia			
Yes		4	5.26
No		72	94.74
Hospitalisation for COVID-19 [n (%)]		5	6.58
Drug therapy during acute infection [n (%)]			
Antibiotics		14	18.42
Inhaled Corticosteroids		2	2.63
Systemic Corticosteroids		4	5.26
NSAID or paracetamol		49	64.47
Hydroxychloroquine		8	10.53
Tocilizumab		1	1.32
Antiviral		2	2.63
Anticoagulant		3	3.95
Other		7	9.21
None		22	28.95

Abbreviations: NSAID= Non-steroidal anti-inflammatory drug.

Supplementary Figures

Figure S1. Percentage of subjects with and without persistent symptoms at 1 year after SARS-CoV2 infection, out of a total of n=76 HCWs.

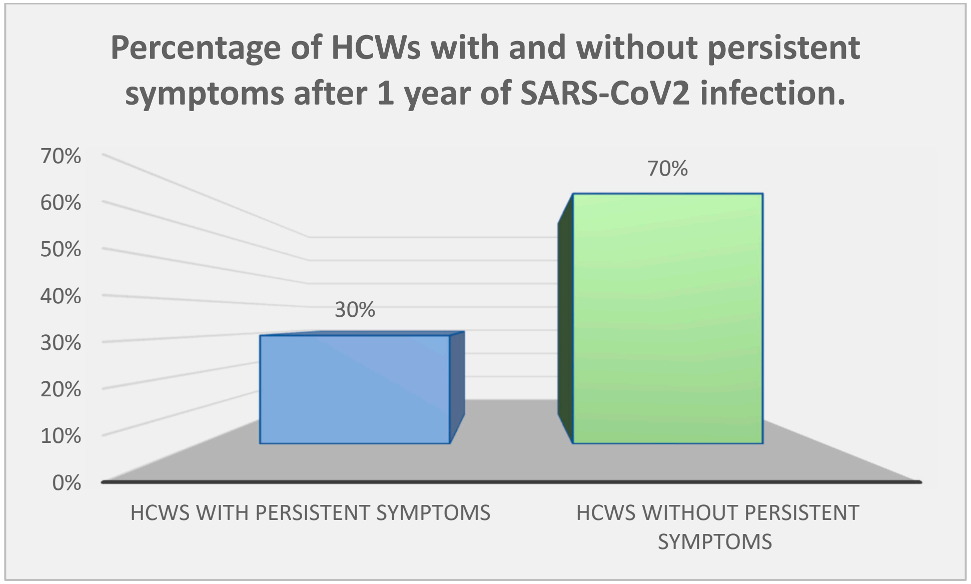

The figure on the right shows the percentage of HCWs with persistent symptoms approximately 1 year after SARS-CoV2 infection, which is about 30% (blue histogram); on the left, the percentage of healthcare workers without persistent symptoms, which is 70% (green histogram).

Figure S2. Correlation curves between NC (A) and IS DNAmAge (B) or IS TL (C) with chronological age of HCWs COVID19 survivors.

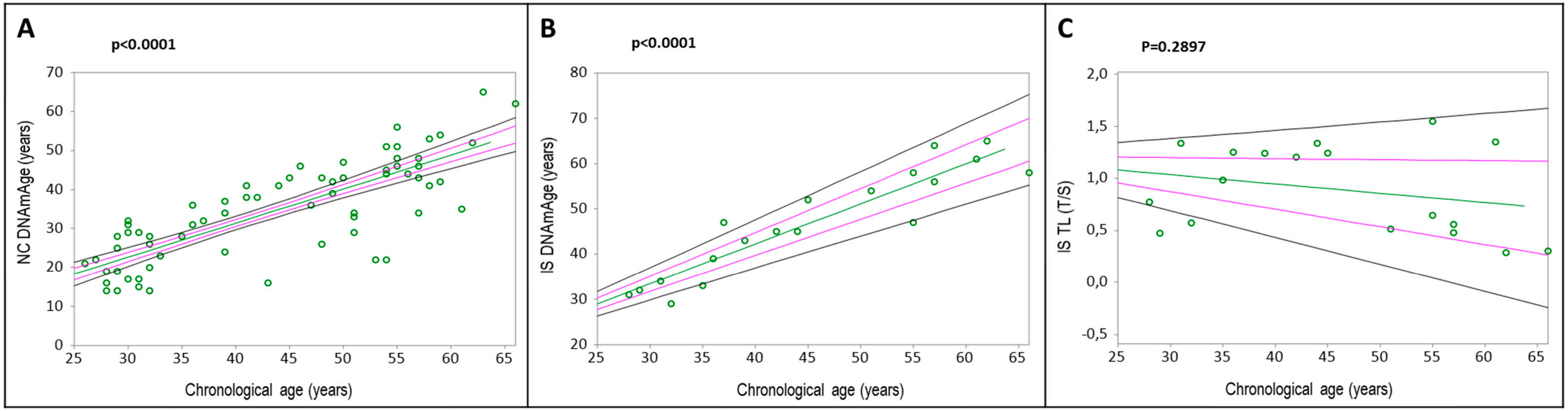

In (A), a simple linear regression plot shows the correlation between NC DNAmAge (years) and chronological age of n=70 HCWs COVID19 survivors [correlation coefficient (r) = 0.8015; two-sided $p < 0.0001$], while in (B), simple linear regression linear regression plot showing the correlation between IS DNAmAge (years) and chronological age of n=19 HCWs COVID19 survivors [correlation coefficient (r) = 0.9279; two-sided $p < 0.0001$]. In (C), a simple linear regression plot shows the correlation between IS TL and chronological age [correlation coefficient (r) = -0.2640; two-sided $p = 0.2897$]. Mean, standard error (SE), and 95% coefficient intervals (CI) are represented as green, pink, and black lines, respectively.

Figure S3. Correlation curves between blood leukocytes DNAmAge and NC DNAmAge (A) or IS DNAmAge (B), and blood leukocytes TL and IS TL (C) of HCWs COVID19 survivors.

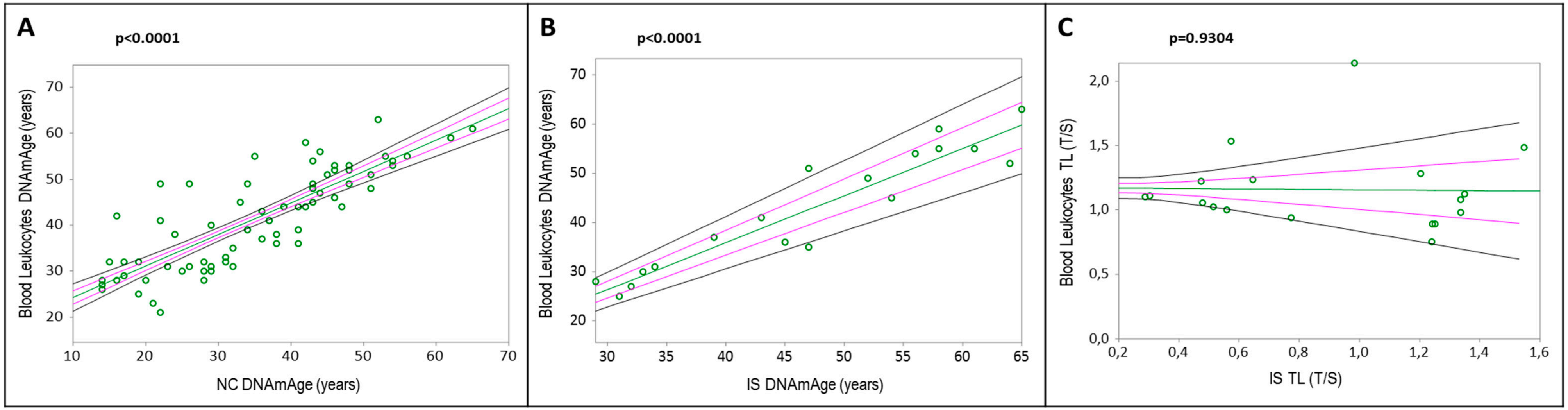

In (A), a simple linear regression plot shows the correlation between blood leukocytes DNAmAge (years) and NC DNAmAge (years) of n=70 HCWs COVID19 survivors [correlation coefficient (r) = 0.8207; two-sided p < 0.0001], while in (B), simple linear regression linear regression plot showing the correlation between blood leukocytes DNAmAge (years) and IS DNAmAge (years) of n=19 HCWs COVID19 survivors [correlation coefficient (r) = 9353; two-sided p < 0.0001]. In (C), a simple linear regression plot shows the correlation between between blood leukocytes TL (T/S) and IS TL (T/S) [correlation coefficient (r) = -0.0222; two-sided p = 0.9304]. Mean, standard error (SE), and 95% coefficient intervals (CI) are represented as green, pink, and black lines, respectively.
